# Low-Dose, Post-Storage Dancong Tea Attenuates Hydroalcohol-Induced Gastric Damage via Modulation Antioxidant and Anti-Inflammatory Pathways

**DOI:** 10.3390/foods14162797

**Published:** 2025-08-12

**Authors:** Huanwei Jian, Ruohong Chen, Lingli Sun, Qiuhua Li, Junxi Cao, Xingfei Lai, Zhenbiao Zhang, Suwan Zhang, Mengjiao Hao, Shili Sun, Zhongzheng Chen

**Affiliations:** 1 Guangdong Provincial Key Laboratory of Nutraceuticals and Functional Foods, College of Food Science, South China Agricultural University, 483 Wushan Street, Tianhe District, Guangzhou 510642, China; 15915931651@163.com; 2 Tea Research Institute, Guangdong Academy of Agricultural Sciences/Guangdong Provincial Key Laboratory of Tea Plant Resources Innovation & Utilization, Guangzhou 510640, China; chenruohong@gdaas.cn (R.C.); sunlingli@tea.gdaas.cn (L.S.); liqiuhua@tea.gdaas.cn (Q.L.); caojunxi@tea.gdaas.cn (J.C.); laixingfei@gdaas.cn (X.L.); zhangzhenbiao@gdaas.cn (Z.Z.); swzhang1502@163.com (S.Z.); haomengjiao@gdaas.cn (M.H.)

**Keywords:** gastric injury, new tea, stored tea, low-dose, inflammatory, antioxidant

## Abstract

Dancong tea is a representative type of oolong tea typically stored for over six months before sale to reduce gastrointestinal irritation. The effects and mechanisms of this storage on gastrointestinal damage remain unclear. Therefore, this study investigated hydrochloric acid and ethanol (HCl/EtOH)-induced gastric injury in mice. The results indicate that six-month-stored Dancong tea (OldT) alleviated gastric injury at low doses but showed no protective effect at high doses; in fact, high-dose OldT exacerbated injury. In contrast, fresh tea (NewT) aggravated gastric injury at both low and high doses. Hematoxylin and eosin (H&E) staining revealed that low-dose OldT significantly attenuated gastric histopathological injury. Mechanistically, low-dose OldT reduced injury via antioxidant and anti-inflammatory pathways (Nrf-2/HO-1 activation and NF-κB inhibition), and inhibiting lipid peroxidation, reactive oxygen species (ROS), nitric oxide (NO), and inflammatory mediators (iNOS, COX-2, IL-6, TNF-α). These findings suggest that storage reduces the gastrointestinal irritant properties of fresh Dancong tea, providing a scientific basis for industrial practice and guiding consumption.

## 1. Introduction

Tea is one of the top three non-alcoholic beverages globally and enjoys significant popularity [1]. Oolong tea is classified as semi-fermented, with a degree of fermentation that lies between that of green tea and black tea. The production process involving sun-greening and shaking greening imbues oolong tea with its unique aroma and flavor [2]. Dancong tea, as a prominent representative, offers notable qualities including a high aroma, resistance to brewing, and various health benefits, with studies indicating its efficacy in anti-inflammatory responses and weight management [3].

The primary processing of oolong tea involves plucking, sun-withering, leaf-tossing, fixation, rolling, and drying, followed by secondary processing steps including sorting, re-roasting, packaging, and storage [4]. As a representative oolong variety, Dancong tea is typically aged for weeks to months prior to market release [5]. Recent studies have highlighted dynamic changes in bioactive components, quality attributes, and antimicrobial efficacy during Dancong tea storage [6,7], while traditional knowledge attributes gastrointestinal symptom relief (e.g., diarrhea mitigation) to aged oolong consumption. However, the potential of stored Dancong tea to alleviate acute gastric injury remains unexplored.

Recent advancements in food bioactives and gastrointestinal health have highlighted the intricate interactions between phytochemical composition, structural attributes, and biological function. For instance, structural modifications of tea constituents during processing or storage can profoundly influence their bioactivity. Liang et al. (2023) demonstrated how resveratrol’s structural modulation under thermal stress alters its bio-efficacy, reinforcing the notion that thermal or temporal changes in bioactives can enhance or suppress physiological outcomes [8]. In a related context, Li et al. (2025) explored the nanoscale characteristics of black tea infusions, revealing how compositional features modulate organoleptic and functional properties via structural aggregation [9]. These insights suggest that the post-processing evolution of tea, such as that occurring during Dancong tea storage, may lead to the formation of novel bioactive complexes with distinct biological effects. Moreover, the gastrointestinal system remains a critical target for dietary interventions, as multiple studies emphasize the relevance of inflammation and oxidative stress pathways in mucosal damage. Compounds such as celastrol and graveoline have been shown to attenuate gastrointestinal and hepatic injury by modulating inflammatory signaling axes, including FOXA1/CLDN4 and JAK1/STAT3, respectively [10,11]. These findings underscore the importance of identifying naturally occurring phytochemicals or processed products—like stored Dancong teas—that might achieve similar protective effects via the Nrf-2/HO-1 or NF-κB pathways. Additionally, polysaccharide-rich extracts, like those derived from *Rubus chingii* fruits, have demonstrated efficacy in regulating intestinal absorption and barrier function, indicating the broader relevance of plant-derived components in digestive health [12]. Together, these studies support the hypothesis that storage-induced transformations in Dancong tea may not only modulate chemical profiles but also influence its ability to mitigate gastric injury through anti-inflammatory and antioxidant mechanisms. Critically, the timeline for these transformations remains underexplored; thus, we selected a 6-month storage period based on preliminary data indicating significant stabilization of key bioactive compounds while aligning with traditional aging practices. As new tea undergoes aging, polyphenols, alcohols, and aldehydes gradually diminish due to air oxidation. This process leads to the formation and accumulation of tea pigments [13]. This alteration may render Dancong tea less irritating to the gastrointestinal mucosa; however, this claim has not been substantiated by research. Furthermore, due to the dual nature of tea components regarding dosage effects on the gastrointestinal tract, it is crucial to examine how varied dosages of stored Dancong tea impact gastric health to guide its scientific utilization.

The gastrointestinal (GI) system, primarily responsible for food digestion and nutrient absorption, encompasses the stomach, small intestine, and large intestine [14]. Gastrointestinal injuries often result in the loss of epithelial mucus, mucosal damage, and inflammation [15]. To rigorously evaluate the gastroprotective potential of stored Dancong tea, we employed the hydrochloric acid–ethanol model—a well-established method for inducing acute gastric injury that reliably recapitulates oxidative and inflammatory stress pathways. The hydrochloric acid–ethanol model is a widely utilized method for inducing gastric injury, noted for its simplicity and brevity of the modeling cycle [16]. Given that inflammation and oxidative stress (mediated by pathways such as NF-κB and Nrf-2/HO-1) are pivotal contributors to gastric mucosal injury, alleviating these factors can be instrumental in mitigating gastric damage [17].

Here, we hypothesize that aging Dancong tea for six months will reduce its gastrointestinal irritancy. Using a hydrochloric acid/ethanol-induced gastric injury model in mice, we test whether stored Dancong tea (OldT) protects gastric mucosa better than fresh tea (NewT), and elucidate the underlying antioxidant and anti-inflammatory mechanisms. We focus on Lingtou Dancong tea, a widely cultivated oolong tea variety in Guangdong, China [2], to provide insights into proper storage and consumption practices for gastrointestinal health benefits.

## 2. Materials and Methods

### 2.1. Tea Sample Preparation

All Dancong tea leaf samples were harvested in the fall of 2023 from “China Lingtou Dancong Tea Township” in Raoping County, Chaozhou City, Guangdong Province. The tea leaves were standardized to consist of pairs of young leaves and were then processed into tea. Processing involved blending, killing, withering, kneading, and roasting to produce Dancong tea. The processed tea leaves were divided into two batches: one batch was immediately frozen at −80 °C for six months to preserve the characteristics of freshly processed tea (a method adapted from preservation protocols in tea metabolite studies [18]), while the other batch was stored under ambient conditions (25 °C, 60% relative humidity) for six months to simulate conventional storage.

For phytochemical extraction, 50 g of ground tea leaves from each batch were mixed with pure water at a 1:20 ratio (*w*/*v*) and subjected to hot water extraction at 95 °C for 45 min for analytical extract preparation (not simulating a typical cup of tea). The resulting extracts were centrifuged at 8000× *g* for 15 min to remove insoluble residues. The supernatants were vacuum-concentrated and lyophilized to obtain freeze-dried tea extract powders. These powders, derived from freshly processed tea leaves and aged tea leaves, were defined as NewT and OldT, respectively. Among them, the extraction rate of new tea was 35.72%, and that of old tea was 35.23%. The NewT and OldT samples were subsequently used for animal treatment experiments to evaluate their effects on gastric injury.

### 2.2. Tea Sample Characterization

The biochemical components were analyzed according to previously established methods [19]. Briefly, the content of polyphenols was measured using the Folin phenol method (GB/T 8313-2018 [20]), free amino acids were quantified by the ninhydrin method (GB/T 8314-2013 [21]), theaflavins, thearubigins, and theabrownins were measured with the People’s Republic of China Agricultural Industry Standard (NY/T 3675-2020 [22]), and total soluble sugar content was measured by the anthrone–sulfuric acid colorimetric assay. The contents of caffeine and catechins were determined by high-performance liquid chromatography (HPLC). The NewT and OldT samples were extracted with 70% (*v*/*v*) methanol in a water bath at 70 °C for 10 min. An Agilent column (5 μm, 250 × 4.6 mm) was used for chromatographic separation. Mobile phase A was double-distilled water, phase B was methanol, phase C was 0.05% (*v*/*v*) phosphoric acid, and phase D was acetonitrile. Gradient elution was performed at the flow rate of 1 mL/min, the sample volume was 10 μL, and the UV detection wavelength was 278 nm. The percentage composition of bioactive compounds (e.g., polyphenols, flavonoids, theaflavins) was calculated as the basis for percentages (dry weight normalization). The biochemical components of NewT and OldT samples are summarized in Table 1 and Table 2.

### 2.3. Establishment of Mouse Model of Gastric Mucosal Injury

Male ICR mice (to avoid estrous cycle variability), aged six weeks, were procured from Dienen Genetic Technology Co., Ltd. (Guangzhou, Guangdong, China). They were maintained in a 12 h light/dark cycle at 60–70% humidity and at room temperature (22 ± 2 °C), with ad libitum access to food and water. After one week of acclimatization, mice were randomly allocated into groups (*n* = 10 per group). All mice received daily oral gavage for three consecutive days: (1) normal control (CON), (2) model (MOD), (3) low-dose (200 mg/kg BW) new Dancong tea (L-NewT), (4) high-dose (600 mg/kg BW) new Dancong tea (H-NewT), (5) low-dose (200 mg/kg BW) after-storage Dancong tea (L-OldT), and (6) high-dose (600 mg/kg BW) after-storage Dancong tea (H-OldT). CON/MOD groups received an equal volume of saline. The experimental doses were determined using body surface area normalization, corresponding to human-equivalent daily tea consumption of 3–5 g/day (typical intake) and 10–15 g/day (potential high intake), respectively. This dose range reflects both routine tea-drinking scenarios and exploratory therapeutic applications.

Gastric mucosal injury was induced by a single gavage of 0.4 M HCl and 60% ethanol in all animals except the control group. After modeling, the drug/extract was administered for three consecutive days in the treatment group, while the control and model groups received equal amounts of saline. Two hours post-final treatment, the mice were sacrificed, and blood, stomach, and liver tissues were harvested for further analysis. The stomachs were immediately excised, cut along the greater curvature, photographed, and weighed. One portion was fixed in 4% paraformaldehyde, while the other was rapidly frozen and stored at −80 °C. All experiments adhered to the Guide for the Care and Use of Laboratory Animals, with approval granted by the Animal Care and Welfare Committee of the Tea Research Institute of the Guangdong Academy of Agricultural Sciences (Serial No. 2024016).

### 2.4. Macroscopic Assessment of Gastric Mucosal Damage

The gastric mucosal ulcer index (UI) was calculated based on the count and diameter of erosions using the formula: UI = Σ[(A × 1) + (B × 2) + (C × 3)], where A, B, and C correspond to the number of lesions measuring ≤1 mm, 1–3 mm, and >3 mm, respectively. Mean scores for each group were calculated to gauge the extent of mucosal injury.

### 2.5. Histologic Staining

Fixed gastric or intestinal tissues were sectioned into 5 mm blocks and embedded in paraffin. Sections of 5 μm were prepared using a paraffin slicer (Leica, Zurich, Switzerland) and subjected to deparaffinization with xylene and a series of ethanol solutions (100%, 95%, 80%, and 70%). Hematoxylin and eosin (H&E) staining was conducted as per standard protocols. Following staining, sections were dehydrated with 95% and 100% ethanol, permeabilized in xylene, and mounted with neutral resin. Stained sections were observed under a light microscope (Olympus, Japan).

### 2.6. Biochemical Assay

Gastric tissue samples were homogenized with 9 volumes of saline using an OMNI Bead Ruptor 24 homogenizer. Each sample analyzed in triplicate technical replicates. The supernatant was collected via centrifugation at 2500 rpm for 10 min at 4 °C. Protein content was quantified using a Pierce BCA Protein Assay Kit (BCA, Thermo, Waltham, MA, USA). Levels of aspartate aminotransferase (AST, C010-2-1, Nanjing JianCheng, Nanjing, China), alanine aminotransferase (ALT, C009-1-2, Nanjing JianCheng, Nanjing, China), malondialdehyde (MDA, A003-1-2, Nanjing JianCheng, Nanjing, China), glutathione (GSH, A006-2-1, Nanjing JianCheng, Nanjing, China), catalase (CAT, A007-1-1, Nanjing JianCheng, Nanjing, China), and superoxide dismutase (SOD, A001-3-2, Nanjing JianCheng, Nanjing, China) were assessed using specific assay kits, following manufacturer’s guidelines. Additionally, nitric oxide (NO, MM-0658M1, Meimian, Wuhan, China), ROS (MM-43700M1, Meimian, Wuhan, China), and PGE2 (MM-46884M1, Meimian, Wuhan, China) levels were quantified with appropriate ELISA kits.

### 2.7. Immunohistochemistry (IHC)

Paraffin sections were treated with 3% H_2_O_2_ to quench endogenous peroxidase activity and subjected to microwave heating in ethylenediaminetetraacetic acid (EDTA) to activate tissue antigens. After blocking with 5% goat serum (SL038, Solarbio, Beijing, China) for 30 min, sections were incubated with primary antibodies, including nuclear factor-erythroid 2-related factor 2 (Nrf-2, Bioss, bs-1074R, Beijing, China, Rabbit, 1:500), heme oxygenase 1 (HO-1, Bioss, bs2075R, Beijing, China, Rabbit, 1:500), interleukin-6 (IL-6, Bioss, bs-0782R, Beijing, China, Rabbit, 1:500), tumor necrosis factor-α (TNF-α, Proteintech, 26405-1-AP, Wuhan, China, Rabbit, 1:500), inducible nitric oxide synthase (iNOS, Proteintech, 22226-1-AP, Wuhan, China, Rabbit, 1:500), cyclooxygenase 2 (COX-2, Proteintech, 12375-1-AP, Wuhan, China, Rabbit, 1:500), and phospho-nuclear factor kappa-B (p-NF-κB, Bioss, bs-0982R, Wuhan, China, Rabbit, 1:500). The following day, sections were probed with corresponding secondary antibodies for one hour, detected using SABC reagent (P0603, Beyotime Biotechnology, Shanghai, China) for 30 min, and developed with DAB (P0203, Beyotime Biotechnology, Shanghai, China) for 2–5 min. Sections were re-stained with hematoxylin (C0107, Beyotime Biotechnology, Shanghai, China) for 90 s, dehydrated through an ethanol gradient, cleared in xylene, and mounted with neutral resin. Quantification was performed by analyzing three random fields per tissue section, with three sections evaluated per mouse, using ImageJ software 1.52a software to calculate mean optical density.

### 2.8. Western Blot

Gastric tissues were homogenized with tenfold volumes of RIPA lysis buffer (RIPA, Beyotime, Shanghai, China), placed on ice for one hour, and centrifuged at 4 °C for 20 min. Protein concentrations were determined via the Pierce BCA Protein Assay Kit (BCA, Thermo, Waltham, MA, USA). Equal protein amounts were denatured in SDS-PAGE loading buffer (containing 4× DTT) for five minutes before being subjected to SDS-PAGE gel electrophoresis. Proteins were transferred to polyvinylidene fluoride membranes, blocked with 5% skim milk for one hour, and subsequently incubated overnight at 4 °C with primary antibodies including Nrf-2 (Bioss, bs-1074R, Beijing, China, Rabbit, 1:1000), HO-1 (Bioss, bs2075R, Beijing, China, Rabbit, 1:1000), IL-6 (CST, #12912S, Danvers,MA, USA, Mouse, 1:1000), TNF-α (Abcam, ab6671, Cambridge, UK, Rabbit, 1:1000), iNOS (Abcam, ab15323, Cambridge, UK, Rabbit, 1:1000), COX-2 (CST, #12282S, Danvers, MA, USA, Rabbit, 1:1000), inhibitor of nuclear factor kappa-B-α (IκB-α, CST, #9242S, Danvers, MA, USA, Rabbit, 1:1000), phospho-inhibitor of nuclear factor kappa-B-α (p-IκB-α, CST, #9246S, Danvers, MA, USA, Rabbit, 1:1000), NF-κB (CST, #8242S, Danvers, MA, USA, Rabbit, 1:1000), p-NF-κB (CST, #3033S, Danvers, MA, USA, Rabbit, 1:1000), B-cell lymphoma-2-associated X protein (Bax, Abcam, AB32503, Cambridge, UK, Rabbit, 1:1000), B-cell lymphoma-2 (Bcl-2, CST, #2870S, Danvers, MA, USA, Rabbit, 1:1000) and β-actin (CST, #3700S, Danvers, MA, USA, Mouse, 1:1000). Membranes were washed with Tris Buffered Saline with Tween-20 six times, followed by incubation with secondary antibodies for 50 min. Bands were visually developed in the dark using the Tanon system, and gray-scale intensity was normalized to β-actin and quantified using ImageJ.

### 2.9. Statistical Analysis

Data were analyzed using GraphPad Prism 9.0 software (GraphPad Software, San Diego, CA, USA). To compare the two groups, Student’s *t*-test was used. For comparisons involving more than two groups, one-way ANOVA with Tukey’s post hoc test was used. Data are presented as the mean ± SD of 10 mice/group (in vivo endpoints), with triplicate technical replicates per sample for biochemical/ELISA assays. * *p* < 0.05 and ** *p* < 0.01 indicated significant differences.

## 3. Results

### 3.1. Biochemical Composition Differences Between NewT and OldT

In this study, we conducted a comparative analysis of the biochemical composition between NewT and OldT. The results are presented in Table 1. Storage induced a significant reduction in parent polyphenols: tea polyphenols decreased by 13.0% (from 18.26% to 15.88% dry weight), while flavonoids declined by 6.7% (0.90% to 0.84%). Conversely, fermentation products increased markedly: thearubigins surged by 115% (1.48% to 3.19%), and theabrownins by 61% (1.04% to 1.67%). Theaflavins, though low in absolute abundance, increased by 19% (0.036% to 0.043%). There was no significant difference in the levels of free amino acids and soluble sugars.

Subsequently, we employed high-performance liquid chromatography (HPLC) to determine the catechin monomer and caffeine contents in NewT and OldT, with the results shown in Table 2. The catechin monomer and caffeine contents in OldT were generally lower than those in NewT, except for catechin (C) content, which was higher in OldT. The contents of epigallocatechin gallate (ECG), epicatechin (EC), and gallic acid (GA) in OldT were lower than those in NewT. These results demonstrate that the compositional content of fresh Dancong tea changes significantly after six months of storage, with a notable increase in the levels of theaflavins, thearubigins, and theabrownin, and a significant decrease in the levels of tea polyphenols, flavonoids, and catechin monomers EC, ECG, and GA.

### 3.2. Effects of Dancong Tea on HCl/EtOH-Induced Gastric Injury in Mice

**Figure 1:**
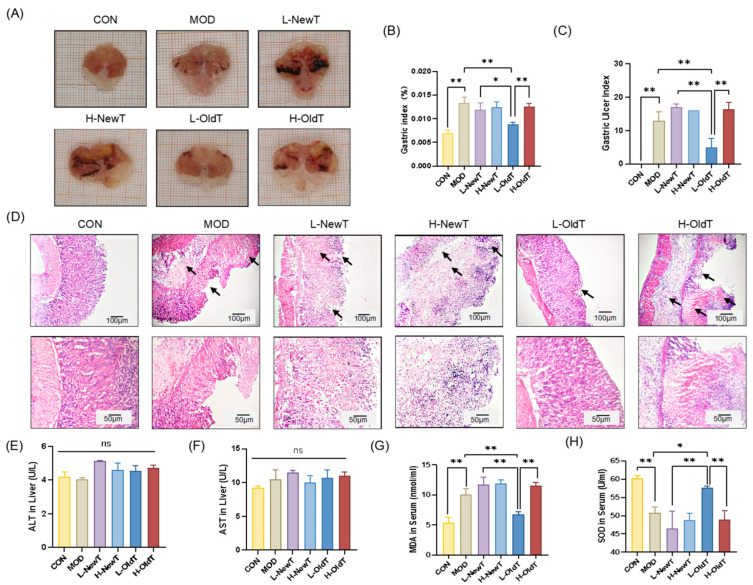
**Effects of freshly prepared Dancong tea and after-storage Dancong tea on HCl/EtOH-induced gastric injury.** (**A**) Anatomical gastric tissue lining in mice. (**B**) Gastric index in mice. (**C**) Gastric mucous membrane damage scoring index. (**D**) H&E-stained sections in mouse stomach (magnification 100×, 200×), Arrows indicate muscular layer edema, epithelial necrosis/sloughing, and inflammatory cell infiltration. (**E**) ALT level in liver tissue (U/L). (**F**) AST level in liver tissue (U/L). (**G**) Serum MDA level (nmol/mL). (**H**) Serum SOD activity (U/mL). Data are expressed as mean ± SD. note: * *p* < 0.05, ** *p* < 0.01.

As shown in Figure 1A and Figure S1, the gastric tissue surface of normal mice was smooth and intact. Mice treated with HCl/EtOH gavage exhibited hemorrhage, burn scarring, and erosive lesions. Compared with the model group, macroscopic observation indicated that the low-dose OldT (L-OldT) group showed visibly reduced ulceration, while lesions persisted in low/high-dose NewT (L/H-NewT) and high-dose OldT (H-OldT) groups. Quantitative analysis (Figure 1B,C) revealed that compared to model (MOD), only L-OldT significantly reduced gastric index (*p* < 0.01) and ulcer score (*p* < 0.01). L-NewT, H-NewT, and H-OldT showed no significant difference compared to MOD (*p* > 0.05). L-OldT gastric index was significantly lower than L-NewT (*p* < 0.05) and H-OldT (*p* < 0.01).

The results of H&E staining of gastric tissues are shown in Figure 1D. The gastric mucosa of the normal group was structurally intact, while the gastric tissues of the model group showed severe necrotic detachment, hemorrhage, and inflammatory cell infiltration. In the low-dose OldT group, only a small amount of gastric mucosal detachment was observed, and the overall situation was not significantly different from that of the normal group. The necrotic area of the gastric epithelial tissue in the low- and high-dose NewT groups and the high-dose OldT group was significantly greater than that in the model group, accompanied by more inflammatory cell infiltration. Additionally, as shown in Figure 1E,F, there was no significant difference (*p* > 0.05) in ALT and AST levels in the liver tissues of the mice in each group, indicating that the tea dose was not hepatotoxic. As shown in Figure 1G,H, compared with the normal group, the serum MDA level was significantly higher, and serum SOD activity was significantly lower in the model group. Compared with the model group, there were no significant differences (*p* > 0.05) in serum MDA levels and SOD activity between the low- and high-dose NewT groups and the high-dose OldT group. However, serum MDA was significantly decreased by 21.70% (*p* < 0.01), and serum SOD activity was significantly increased by 13.45% (*p* < 0.05) in the low-dose OldT group vs. MOD group. The serum MDA level in the low-dose OldT group was significantly lower than that in the high-dose OldT and low-dose NewT groups (*p* < 0.01), and the serum SOD level was significantly higher than that in the high-dose OldT and low-dose NewT groups (*p* < 0.01). This suggests that freshly prepared Dancong tea exacerbates gastric mucosal damage, which is significantly improved by proper storage, particularly at low doses of Dancong tea (3–5 g/d), while high doses (10–12 g/d) exacerbate it.

### 3.3. Effect of Dancong Tea on Oxidative Stress in Mice with HCl/EtOH-Induced Gastric Injury

**Figure 2:**
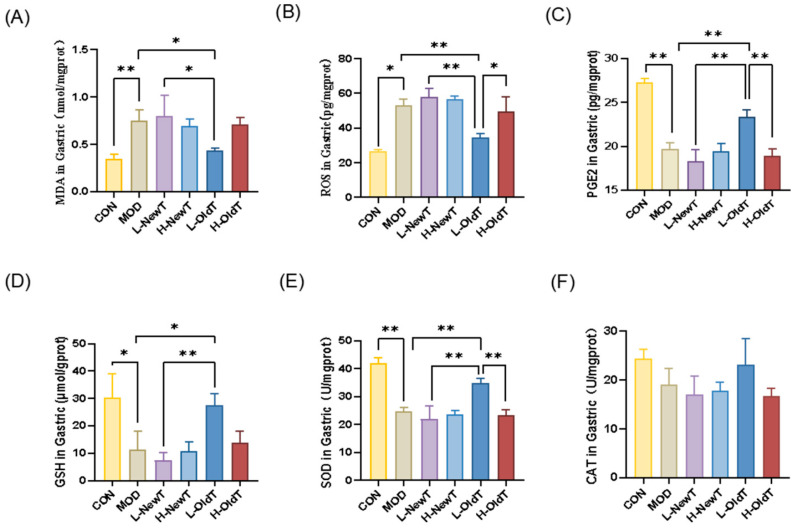
**L-OldT alleviates oxidative stress in mice with HCl/EtOH-induced gastrointestinal injury.** (**A**) Gastric tissue MDA content. (**B**) Gastric tissue ROS level. (**C**) Gastric tissue PGE2 level. (**D**) Gastric tissue GSH content. (**E**) Gastric tissue SOD activity. (**F**) Gastric tissue CAT activity (*n* = 3 independent experiments). Data are expressed as mean ± SD. note: * *p* < 0.05, ** *p* < 0.01.

Oxidative stress is considered one of the key factors in gastric mucosal damage [16]. As shown in Figure 2A,B, compared with normal mice, gastric tissues from HCl/EtOH-induced gastric injury mice had significantly higher MDA (*p* < 0.01) and ROS (*p* < 0.05) content. Compared with the model group, there was no significant difference (*p* > 0.05) in MDA and ROS levels in the gastric tissues of the low- and high-dose NewT and high-dose OldT groups, while the low-dose OldT group had a 41% decrease in gastric MDA levels. The gastric MDA and ROS levels in the low-dose OldT group were reduced by 41.72% (*p* < 0.05) and 35.08% (*p* < 0.01) vs. MOD group, respectively, and were lower than those in the high-dose OldT and low-dose NewT groups.

In addition, prostaglandin E2 (PGE2) is an important protective substance for the gastric mucosa [17]. As shown in Figure 2C, compared with the normal group, the gastric PGE2 level in the model group was significantly lower; among the tea administration groups, only the low-dose OldT group showed a significant increase in gastric PGE2 levels compared with the model group, with no significant difference (*p* > 0.05) in the rest of the groups. The gastric PGE2 level in the low-dose OldT group was significantly higher than that in the high-dose OldT group and the low-dose NewT group.

Antioxidant enzymes and components secreted by organisms can scavenge free radicals and reduce oxidative stress levels, thus protecting the gastric mucosal barrier [16]. As shown in Figure 2D–F, HCl/EtOH gavage significantly decreased GSH levels (*p* < 0.05) and SOD activity (*p* < 0.01) in gastric tissues of the model group compared to the normal group, and low-dose OldT treatment significantly reversed these changes, with effects stronger than those of the high-dose OldT and low-dose NewT groups (*p* < 0.01). There was no significant difference (*p* > 0.05) in gastric GSH levels and SOD activity in the other tea groups compared with the model group. CAT levels did not show statistically significant differences between the groups. This result reveals that storage significantly enhances the role of freshly made Dancong tea in alleviating the level of oxidative stress at the site of gastric mucosal damage.

### 3.4. Effect of Dancong Tea on Inflammation in Mice with HCl/EtOH-Induced Gastric Injury

**Figure 3:**
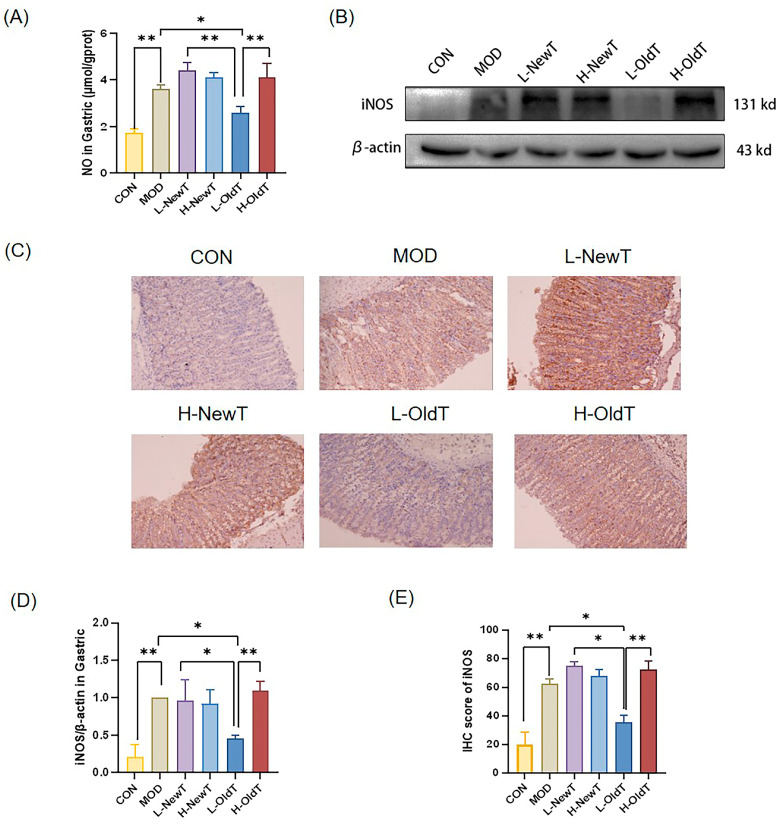
**Inhibition of iNOS expression as well as NO secretion by L-OldT.** (**A**) NO content of gastric tissues. (**B**) Western blot showing the relative expression level of iNOS in gastric tissues. (**C**) Immunohistochemical staining of iNOS in gastric tissues. (**D**) Quantitative analysis of iNOS expression levels by Western blot. (**E**) Quantitative analysis of iNOS expression levels by IHC. Data are expressed as mean ± SD. Note: * *p* < 0.05, ** *p* < 0.01.

In the state of gastric injury, immune cells increase the secretion of nitric oxide (NO), leading to gastric oxidative damage [23]. As shown in Figure 3A, compared with the normal group, the secretion level of NO in gastric tissues of the model group was significantly increased. Compared with the model group, there was no significant difference (*p* > 0.05) in gastric tissue NO levels between the low- and high-dose NewT groups and the high-dose OldT group, whereas the gastric tissue NO level of the low-dose OldT group was significantly reduced by 28.41% (*p* < 0.01), and this effect was significantly stronger than that of the low-dose NewT group (*p* < 0.05) and the high-dose OldT group (*p* < 0.01).

As shown by Western blot and immunohistochemistry in Figure 3B–E, HCl/EtOH gavage significantly upregulated the expression level of iNOS in the gastric tissues of mice. The low-dose OldT treatment for 2 days significantly reversed this change, with an effect significantly stronger than that of the low-dose NewT and high-dose OldT treatments. The expression levels of iNOS in gastric tissues were not significantly different in any of the other tea groups compared to the model group.

**Figure 4:**
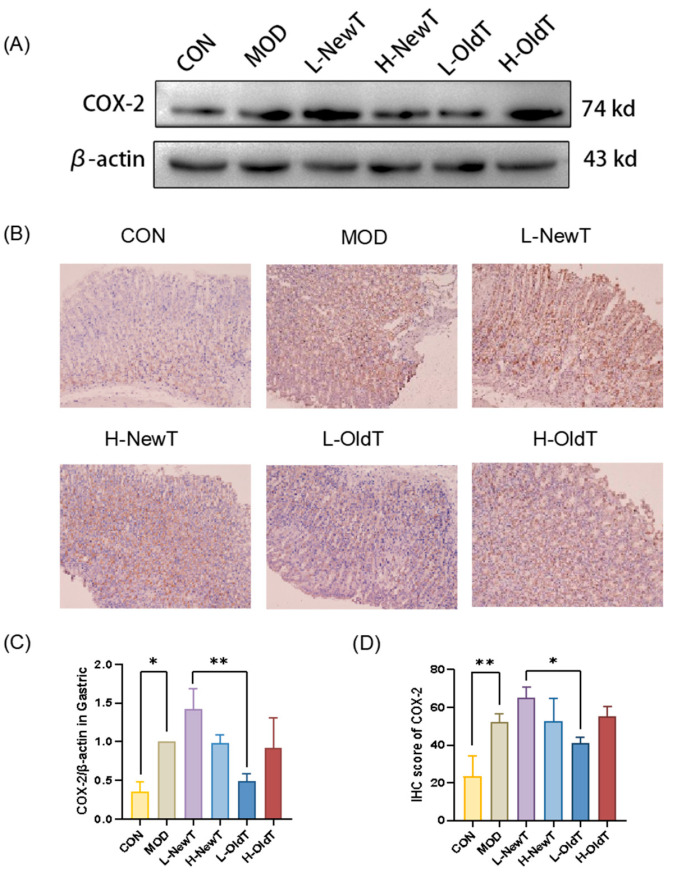
**Inhibition of induced COX-2 expression at gastric injury site by L-OldT.** (**A**) Western blot showing the relative expression level of COX-2 in gastric tissues. (**B**) Immunohistochemical staining of COX-2 in gastric tissues. (**C**) Quantitative analysis of COX-2 expression levels by Western blot. (**D**) Quantitative analysis of COX-2 expression levels by IHC (*n* = 3). Data are expressed as mean ± SD. Note: * *p* < 0.05, ** *p* < 0.01.

COX-2 is an important inflammatory mediator that converts arachidonic acid to prostaglandin analogs, thereby exacerbating inflammation and aggravating peptic ulcers [19]. As shown in Figure 4, compared with the normal group, gastric gavage with HCl/EtOH significantly upregulated the expression level of COX-2 in the gastric tissues of the model group, the high-dose OldT group, and the low- and high-dose NewT groups. After treatment with a low-dose OldT, the expression level of COX-2 in the gastric tissues was downregulated to a level not significantly different from that of the normal group. The COX-2 expression level in the low-dose OldT group was significantly lower than that of the low-dose NewT group.

**Figure 5:**
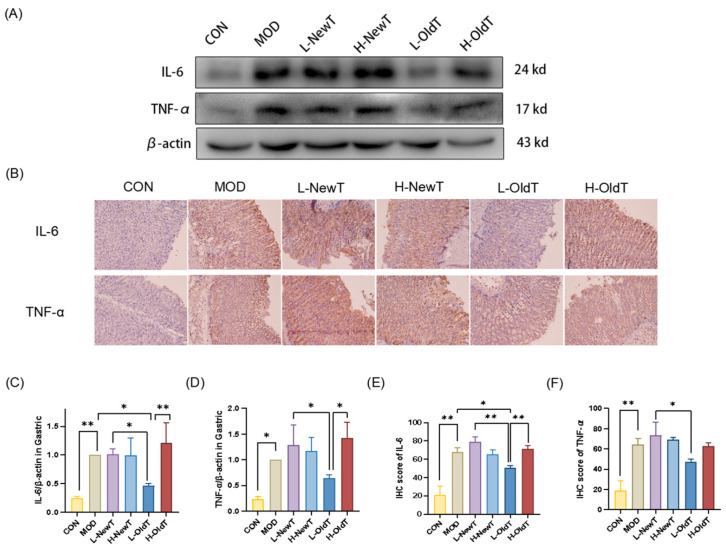
**L-OldT inhibits the induced expression of pro-inflammatory factors IL-6 and TNF-α at the site of gastric injury.** (**A**) Western blot showing the relative expression levels of IL-6 and TNF-α in gastric tissues. (**B**) Immunohistochemical staining of IL-6 and TNF-α in gastric tissues. (**C**,**D**) Quantitative analysis of IL-6 and TNF-α expression levels by Western blot. (**E**,**F**) Quantitative analysis of IL-6 and TNF-α expression levels by IHC. Data are expressed as mean ± SD. Note: * *p* < 0.05, ** *p* < 0.01.

As shown in Figure 5, the expression levels of IL-6 and TNF-α in gastric tissues of the model group were significantly upregulated compared to those of the normal group. There was no significant difference (*p* > 0.05) in the expression levels of IL-6 and TNF-α in gastric tissues between the low- and high-dose NewT groups and the high-dose OldT group when compared to the model group. In contrast, the expression levels of IL-6 and TNF-α in gastric tissues of the low-dose OldT group were downregulated compared to the model group, with statistically significant differences in IL-6 expression levels (*p* < 0.05). The downregulation of IL-6 and TNF-α expression levels in the gastric tissues of the low-dose OldT group was significantly stronger than that observed in the low-dose NewT group and the high-dose OldT group. These results indicate that NewT did not ameliorate the abnormal expression of inflammatory mediators in gastric injury, whereas OldT exerted superior anti-inflammatory effects on gastric mucosal injury compared to NewT, except at high doses.

### 3.5. Effects of Dancong Tea on Oxidative Stress, Inflammatory Pathways, and Apoptotic Proteins

**Figure 6:**
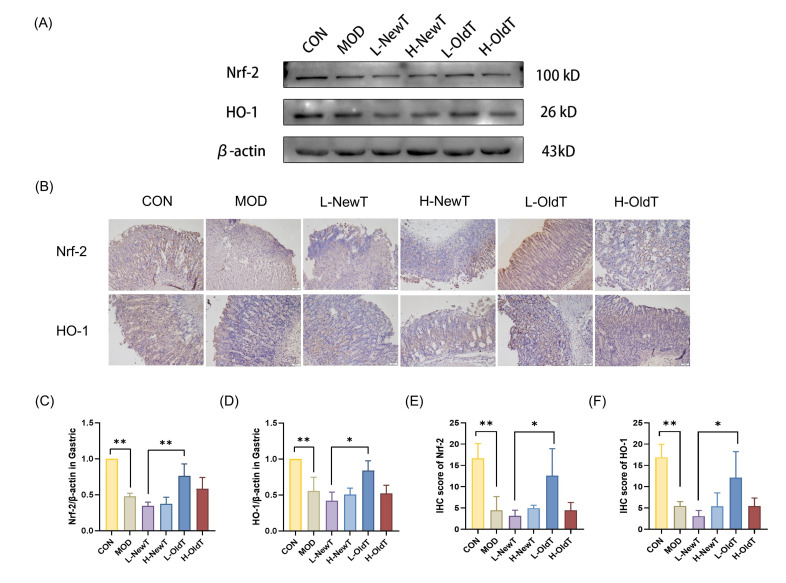
**L-OldT upregulates the expression levels of antioxidant pathway proteins Nrf-2 and HO-1 in the gastric injury site.** (**A**) Western blot showing the relative expression levels of Nrf-2 and HO-1 in gastric tissues. (**B**) Immunohistochemical staining of Nrf-2 and HO-1 in gastric tissues. (**C**,**D**) Quantitative analysis of Nrf-2 and HO-1 expression levels by Western blot. (**E**,**F**) Quantitative analysis of Nrf-2 and HO-1 expression levels by IHC. Data are expressed as mean ± SD. Note: * *p* < 0.05, ** *p* < 0.01.

To further explore the molecular regulatory mechanisms of the antioxidant activity of low-dose Dancong tea, the expression levels of Nrf-2 and HO-1 were analyzed by Western blot and immunohistochemical detection. As shown in Figure 6, compared with the normal group, the expression levels of gastric Nrf-2 and HO-1 were significantly downregulated in the model group, the low- and high-dose NewT groups, and the high-dose OldT group. However, in the low-dose OldT group, the expression levels of these two key proteins in the antioxidant pathway were upregulated to a level not significantly different from that of the normal group, and the expression levels of Nrf-2 and HO-1 in the low-dose OldT group were significantly higher than those in the low-dose NewT group.

**Figure 7:**
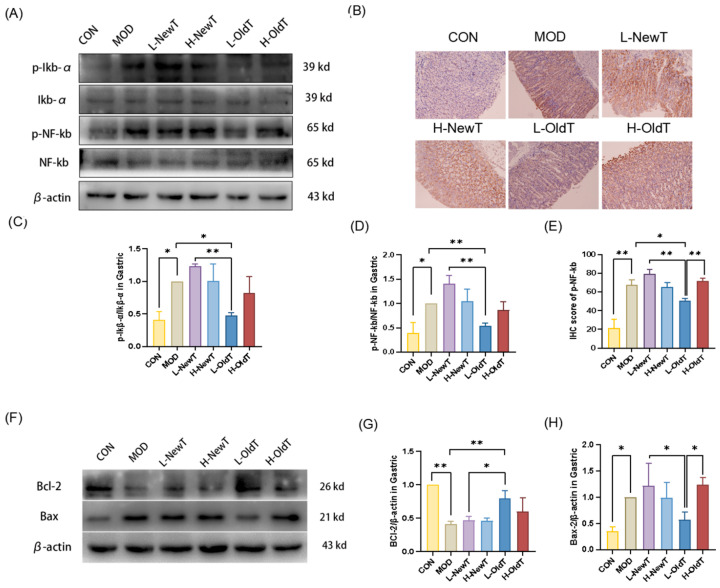
L-OldT downregulates the expression level of inflammatory pathway protein NF-κB p65 and apoptosis proteins at the site of gastric injury. (**A**) Western blot showing the relative expression level of IκB, p-IκB, NF-κB, and p-NF-κB in gastric tissues. (**B**) Immunohistochemical staining of p-NF-κB in gastric tissues. (**C**) Quantification of the relative expression intensity of p-IκB. (**D**) Quantitative analysis of p-NF-κB expression levels by Western blot. (**E**) Quantitative analysis of p-NF-κB expression levels by IHC. (**F**) Western blot showing the relative expression levels of Bax and Bcl-2 in gastric tissues. (**G**,**H**) Quantitative analysis of Bax and Bcl-2 expression levels by Western blot, respectively. Data are expressed as mean ± SD. Note: * *p* < 0.05, ** *p* < 0.01.

As shown in Figure 7A–E, IκB-α and NF-κB phosphorylation levels were significantly increased in gastric tissues of mice after hydroalcoholic gavage compared with those of normal mice. This change was significantly reversed after 2 days of low-dose OldT treatment, with an effect stronger than that of low-dose NewT as well as high-dose OldT. In contrast, the gastric tissues of the low- and high-dose NewT groups and high-dose OldT groups showed a significant increase in the phosphorylation of IκB-α and NF-κB, with effects stronger than those of the low- and high-dose NewT groups.

As shown in Figure 7E–G, the expression level of the anti-apoptotic protein Bcl-2 in gastric tissues was highly significantly downregulated in the model group compared with the normal group. Compared with the model group, there was no significant difference (*p* > 0.05) in the expression level of Bcl-2 in the high-concentration Dancong tea group, as well as in the low- and high-dose NewT groups. However, the expression level of Bcl-2 was significantly upregulated in the low-dose OldT group (*p* < 0.05). Moreover, compared with the normal group, ethanol hydrochloride significantly upregulated the expression level of the pro-apoptotic protein Bax in the model group, high-dose OldT group, and low- and high-dose NewT groups. In contrast, the expression level of Bax in gastric tissues was restored to a level not significantly different from that of the normal group after treatment with low-dose OldT. This suggests that compared to new tea, properly stored Dancong tea significantly modulates the expression of antioxidant as well as anti-inflammatory-related pathways, which in turn reduces oxidative stress and inflammation-induced programmed apoptosis, with the exception of high-dose tea.

### 3.6. Correlation Analysis Between Bioactive Components of Dancong Tea and Indicators Related to Gastric Injury

**Figure 8:**
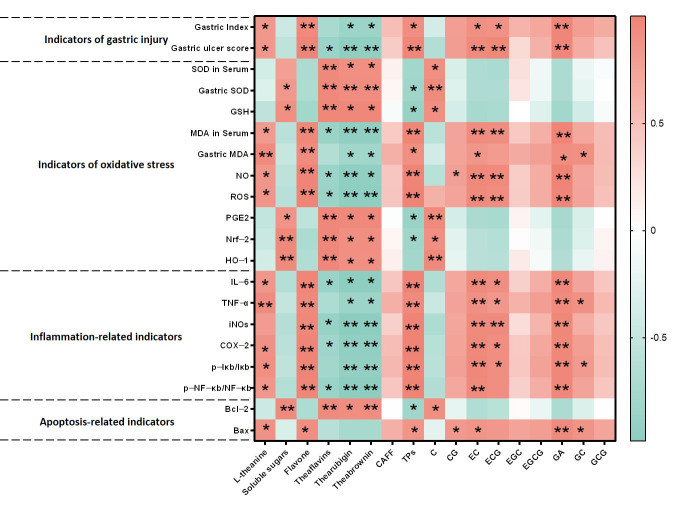
**Analysis of the correlation between quality changes and gastric mucous membrane damage-related indexes of Dancong tea**. Note: * *p* < 0.05, ** *p* < 0.01.

To identify the chemical basis for the reduced gastric mucosal irritation of stored Dancong tea, Pearson correlation analysis was performed between its bioactive components (OldT vs. NewT) and gastric injury biomarkers (Figure 8). The results demonstrated that L-theanine, flavonoids, tea polyphenols, EC, ECG, and GA showed significant positive correlations with gastric mucosal injury severity, oxidative stress markers (reduced SOD and GSH), and inflammatory/apoptotic indices (elevated NF-κB, decreased Bcl-2). Tea polyphenols levels were positively correlated with ulcer severity and NF-κB, but negatively with SOD, PGE2, and Nrf-2. In contrast, theaflavins and thearubigins correlated negatively with ulcer severity, while positively correlating with antioxidant pathway proteins (Nrf-2, HO-1), anti-apoptotic protein Bcl-2, and protective metabolites (SOD, GSH, PGE2), suggesting these components as candidate protective agents warranting further validation. Soluble sugars and residual catechins also positively correlated with these protective factors.

Storage duration induced marked compositional changes: tea polyphenols, flavonoids, EC, ECG, and GA decreased significantly, whereas theaflavins and thearubigins increased substantially (Table 1 and Table 2), suggesting that catechin conversion to theaflavins/thearubigins enhances protection via Nrf-2/HO-1/NF-κB modulation.

## 4. Discussion

Dancong tea is one of the representative teas of oolong tea and has health benefits such as anti-inflammatory properties and weight loss [3]. However, fresh Dancong tea exhibits gastrointestinal irritancy, prompting traditional storage practices to reduce this effect [2]. The mechanisms and chemical basis underlying this storage-mediated reduction in irritancy remain incompletely understood. This study employed a murine HCl/EtOH-induced gastric injury model to investigate the gastroprotective effects of Dancong tea across different storage durations (fresh vs. 6-month stored) and doses (200 mg/kg vs. 600 mg/kg BW/d, approximating human intakes of 3–5 g/d and 10–12 g/d, respectively). Our central finding is that L-OldT significantly alleviated gastric mucosal damage, whereas H-OldT and both doses of NewT failed to confer protection and often exacerbated injury (Figure 1). Critically, this establishes a clear interdependence between storage duration and dosage: only the combination of aging (6 months) and low-dose intake (200 mg/kg) conferred gastroprotection.

**Figure 9:**
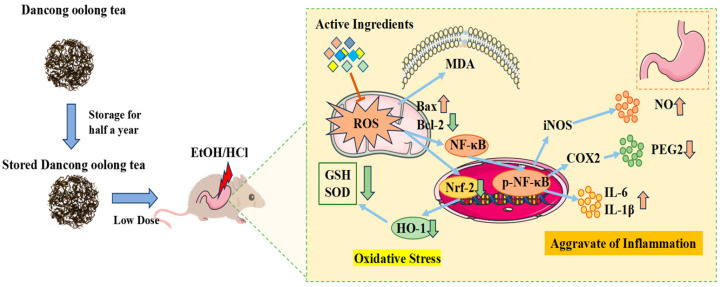
**L-OldT alleviates gastric mucosa injury through anti-inflammatory and antioxidant effects**. Upward arrows denote upregulated expression levels, while downward arrows indicate downregulated expression.

Mechanistically, low-dose properly stored Dancong tea inhibited lipid peroxidation, ROS and NO production, and the expression of inflammatory factors IL-6 and TNF-α, along with inflammatory mediators iNOS and COX-2 through the modulation of the Nrf-2/HO-1 and NF-κB pathways, significantly reducing the deterioration of gastric mucosal damage. In contrast, high doses of Dancong tea and both low and high doses of NewT did not show such effects (Figure 9). Therefore, moderate consumption of stored Dancong tea (≤200 mg/kg BW/d) can help alleviate gastrointestinal damage in individuals with gastrointestinal discomfort, while high-dose tea (≥600 mg/kg BW/d) and new tea are not advisable.

Traditional wisdom suggests that the stimulant properties of NewT are mainly due to its high content of tea polyphenols, aldehydes, and caffeine. However, after a period of storage, these irritants are reduced and more stable bioactive components are formed [13]. Compared to fresh Dancong tea, after-storage Dancong tea has significantly lower contents of tea polyphenols, flavonoids, and catechin monomers such as ECG, EC, and GA, along with significantly higher contents of theaflavins, thearubigins, and theafucoxanthins (Table 1 and Table 2), which aligns with a related study [13]. In a gastric injury mouse experiment, the model group exhibited severe gastric mucosal injury, including gastric erosion, mucosal necrosis, and epithelial cell loss after ingesting HCl and ethanol for 2 days [16]. This study revealed that only low-dose post-storage Dancong tea (200 mg/kg) alleviated ethanol/HCl-induced gastric mucosal injury, reducing necrosis, edema, and inflammation. Conversely, high-dose regular Dancong tea (600 mg/kg) and freshly prepared oolong tea (at both doses) exacerbated mucosal damage (Figure 1). The European Food Safety Authority (EFSA) [24] conducted extensive studies demonstrating that daily intake of Epigallocatechin gallate(EGCG) exceeding or equaling 800 mg may induce hepatotoxicity. The recommended single-dose limit for EGCG is ≤300 mg. In our study, the low- and high-dose tea aqueous extracts corresponded to human EGCG exposures of 211.8 mg and 635.5 mg/day, respectively, based on body surface area normalization. While serum ALT and AST levels indicated no hepatotoxicity in mice under experimental conditions (Figure 1E,F), the high-dose group approached risk thresholds. Critically, real-world tea consumption involves multiple infusions of the same leaves, coupled with EGCG’s low bioavailability and instability during digestion [25]. These factors substantially reduce systemic EGCG exposure, rendering moderate tea consumption—particularly avoiding concentrated brews—both safe and beneficial. This aligns with our findings that low-dose aged Dancong tea alleviates gastric injury, whereas excessive doses exacerbate mucosal damage.

In this study, the tea polyphenol content of Dancong tea was 18.26%, and the gavage dose of 200 mg/kg BW/d corresponded to an intake of more than 626 mg of tea polyphenols per day in 60 kg gastric-injured individuals, which can exacerbate gastric injury. The polyphenol content of stored Dancong tea was 15.88%, and the low dose was equivalent to less than 545 mg of tea polyphenol per day for 60 kg of people with gastric injury, which is favorable for the repair of gastric injury. Additionally, consuming 10–12 g of Dancong tea at one time also aggravated the gastric injury in mice. Critically, the high-dose model employs fully solubilized extracts to deliver bioactives equivalent to consuming 10–12 g of tea leaves in a single bolus—a scenario divergent from traditional tea preparation where 3–5 g of leaves are steeped multiple times in hot water and consumed gradually over hours. While dosage extrapolation to humans is conceivable, we emphasize that these are only approximations, and human studies are required. However, in daily life, 3–5 g of tea should be brewed at one time and consumed in several portions. Therefore, it is recommended that patients with gastric injury avoid high-dose tea and that regular tea consumption is beneficial for the stomach. Conversely, for other symptoms (e.g., obesity), new tea as well as high concentrations (above 600 mg/kg BW/d) of Dancong tea may provide significant relief [3].

Oxidative stress is one of the major drivers of gastric injury [23]. Tea, as a natural antioxidant, acts as a scavenger of ROS and is considered to play a significant role in the relief of gastric ulcers [26]. The results of the present study showed that low-dose OldT inhibited gastric injury-induced ROS and lipid peroxide accumulation via Nrf-2/HO-1 pathway activation, whereas high-dose or freshly prepared oolong tea lacked antioxidant efficacy and exacerbated oxidative stress (Figure 1 and Figure 6). These findings align with related studies on the antioxidant activity of tea [1,17], reflecting the importance of suitable storage for the antioxidant regulatory capacity of Dancong tea.

Imbalances in the antioxidant system exacerbate ROS accumulation, triggering NF-κB activation via IκB-α dissociation and NF-κB p65 release, thereby upregulating pro-inflammatory factors (e.g., TNF-α, IL-6) and amplifying inflammation. This study demonstrated that stored Dancong tea, unlike NewT, suppressed NF-κB signaling and downstream cytokines (Figure 5 and Figure 7), consistent with reports of NF-κB inhibition alleviating gastric injury. Additionally, L-OldT downregulated iNOS and COX-2 expression (linked to excess NO and PGE2 depletion [27]), restoring NO/PGE2 balance (Figure 2, Figure 3 and Figure 4), akin to findings with fermented soybeans. Crosstalk between NF-κB and Nrf-2/HO-1 pathways likely underpins this effect, as NF-κB modulation interacts with Nrf-2-mediated antioxidant defenses. Furthermore, L-OldT reduced apoptosis by restoring the Bcl-2/Bax balance disrupted by ROS, paralleling studies on tea-mediated hepatoprotection and gastric apoptosis inhibition. Thus, stored Dancong tea mitigates gastric injury by blocking ROS-driven Nrf-2/NF-κB crosstalk, alleviating oxidative stress, inflammation, and apoptosis. However, the causative role of these pathways in attenuating gastric injury remains to be mechanistically validated through further inhibitor experiments or genetic knockdown animal models.

Our correlation analyses reveal a dual role of tea components in gastric mucosal protection, modulated by storage-induced chemical transformations. NewT containing elevated levels of tea polyphenols, L-theanine, EC, ECG, and GA exhibited paradoxical pro-oxidative and pro-inflammatory effects at high concentrations, as evidenced by their positive correlations with gastric injury biomarkers (NF-κB activation, Bax elevation) and negative associations with antioxidant defenses (SOD, GSH) and Nrf-2 expression (Figure 8). This concentration-dependent duality aligns with previous findings where low-dose polyphenols demonstrated mucosal protection, while excessive levels exacerbated oxidative damage through redox cycling mechanisms [28,29,30]. The observed dose–response relationship provides a plausible explanation for the gastric irritancy of newly processed Dancong tea.

Correlations between NewT and OldT components (e.g., theaflavins) and Nrf-2/NF-κB expression suggest candidate protective agents; however, definitive mechanistic validation requires future studies using pathway inhibitors or genetic models. These findings are mechanistically supported by Alanazi et al.’s demonstration of theaflavins’ dual regulation of Nrf-2 antioxidant and NF-κB inflammatory pathways in renal protection [31], and Adhikary et al.’s report on theaflavin-mediated gastric ulcer healing through enhanced antioxidant capacity [32]. The structural evolution from monomeric catechins to polymeric pigments during storage appears critical—oxidative polymerization not only increases molecular stability but reduces phenolic hydroxyl exposure, thereby lowering gastrointestinal astringency while preserving redox-modulating capacity [33]. Our quantification of this transformation (Table 1 and Table 2) provides direct evidence for the traditional practice of tea aging in gastrointestinal health preservation. Three key implications emerge from these findings: First, the catechin-theaflavin conversion appears to shift tea’s biological activity from potential irritancy to mucosal protection. Second, the Nrf-2/HO-1/NF-κB axis serves as a pivotal regulatory node connecting chemical composition with gastric outcomes. Third, the time-dependent nature of these transformations underscores the importance of storage duration optimization, though our single timepoint analysis currently limits precise temporal recommendations.

While this study establishes important correlations between chemical transformations and the gastroprotective effects of stored Dancong tea, several limitations warrant acknowledgment. First, the experimental design focused on a single storage timepoint (6 months) and utilized frozen-stored tea as a proxy for NewT—conditions that may not fully replicate commercial aging processes or fresh tea. Second, the HCl/EtOH-induced mouse model reflects acute gastric injury rather than chronic human pathologies (e.g., *H. pylori* infections or non-steroidal anti-inflammatory drugs-induced ulcers), and interspecies differences in mucosal homeostasis limit direct clinical extrapolation. Third, the absence of a positive control (e.g., sucralfate) precludes efficacy benchmarking against established therapeutics. Fourth, human-equivalent dosing (200–600 mg/kg) assumes theoretical bioavailability without clinical validation. Finally, our compositional analysis, though comprehensive, cannot fully resolve storage-driven complexity (e.g., microbial bioconversion versus oxidative polymerization). To address these gaps, we are currently employing high-resolution metabolomics (UPLC-QTOF-MS) to systematically map the dynamic chemical landscape of aging Dancong tea across multiple storage durations. This ongoing work aims to (1) identify novel transformation products beyond the characterized theaflavins/thearubigins, and (2) establish temporal thresholds for the catechin–polymer conversion process. The resulting chemical atlas will enable targeted mechanistic studies to decipher how specific transformation pathways (e.g., oxidative polymerization vs. microbial bioconversion) differentially modulate gastric protective mechanisms through Nrf-2/NF-κB crosstalk. Future studies should prioritize dose–response trials in human cohorts and investigate long-term safety and efficacy.

## 5. Conclusions

In conclusion, freshly prepared Dancong tea exacerbates gastric mucosal damage, while proper storage improves the irritation effects of NewT on gastric mucosa, particularly at low doses, attenuating hydroalcohol-induced gastric damage. This process relies on regulating the Nrf-2/HO-1/NF-κB molecular crosstalk to inhibit lipid peroxidation, production of ROS and NO, and modulate the expression of pro-inflammatory factors IL-6 and TNF-α, along with inflammatory mediators iNOS and COX-2, but can exacerbate gastric injury at high doses. This suggests that patients with gastric injury can beneficially consume moderate amounts of after-storage Dancong tea (3–5 g/day) while avoiding higher doses (10–12 g/day) of high-dose or new teas. This study provides a scientific basis for understanding the storage and processing of Dancong tea and offers guidance for tea consumption among patients with gastrointestinal disorders. However, these results were only obtained at the animal level in this study. Therefore, further studies in human cohorts are warranted to validate these findings.

## Figures and Tables

**Table 1:** Comparison of biochemical content (% dry weight ± SD) between NewT and OldT.

Ingredients	NewT	OldT
Tea polyphenols (%)	18.26 ± 0.28 ^a^	15.88 ± 0.21 ^b^
Free amino acid (%)	1.6 ± 0.02 ^a^	1.62 ± 0.02 ^a^
Soluble sugar (%)	9.89 ± 0.09 ^a^	10.27 ± 0.27 ^a^
Flavonoid (%)	0.90 ± 0.02 ^a^	0.84 ± 0.01 ^b^
Theaflavins (%)	0.036 ± 0.001 ^b^	0.043 ± 0.001 ^a^
Thearubigin (%)	1.481 ± 0.035 ^b^	3.187 ± 0.015 ^a^
Theabrownin (%)	1.038 ± 0.011 ^b^	1.668 ± 0.013 ^a^

Data are presented as the mean of three independent experiments ± SD. Significance accepted at *p* < 0.05. Different superscript letters (a, b) are used to indicate significant differences.

**Table 2:** Comparison of catechin and caffeine contents (% dry weight ± SD) between NewT and OldT.

Ingredients	NewT	OldT
CG (%)	0.13 ± 0.02 ^a^	0.04 ± 0.00 ^a^
ECG (%)	1.68 ± 0.01 ^a^	1.57 ± 0.04 ^b^
GCG (%)	0.20 ± 0.03 ^a^	0.18 ± 0.03 ^a^
EGCG (%)	7.66 ± 0.08 ^a^	7.57 ± 0.09 ^a^
EC (%)	0.52 ± 0.00 ^a^	0.50 ± 0.01 ^b^
C (%)	0.28 ± 0.02 ^a^	0.33 ± 0.01 ^a^
GC (%)	0.26 ± 0.01 ^a^	0.25 ± 0.04 ^a^
EGC (%)	0.14 ± 0.01 ^a^	0.14 ± 0.00 ^a^
GA (%)	0.38 ± 0.00 ^a^	0.37 ± 0.00 ^b^
CAFF (%)	3.12 ± 0.04 ^a^	3.10 ± 0.02 ^a^

Data are presented as the mean of three independent experiments ± SD. Significance accepted at *p* < 0.05. Different superscript letters (a, b) are used to indicate significant differences.

## Data Availability

The original contributions presented in the study are included in the article/Supplementary Material, further inquiries can be directed to the corresponding authors.

## Supplementary Materials

The following supporting information can be downloaded at: https://www.mdpi.com/article/10.3390/foods14162797/s1, Figure S1: Anatomical drawings of representative gastric tissues of each group.
