# Supplementary material for: Low-Dose, Post-Storage Dancong Tea Attenuates Hydroalcohol-Induced Gastric Damage via Modulation Antioxidant and Anti-Inflammatory Pathways
Source: Foods. 2025 Aug 12;14(16):2797. doi: 10.3390/foods14162797 (PMC12385579; doi:10.3390/foods14162797)

Figure.S1 Anatomical drawings of representative gastric tissues of each group.

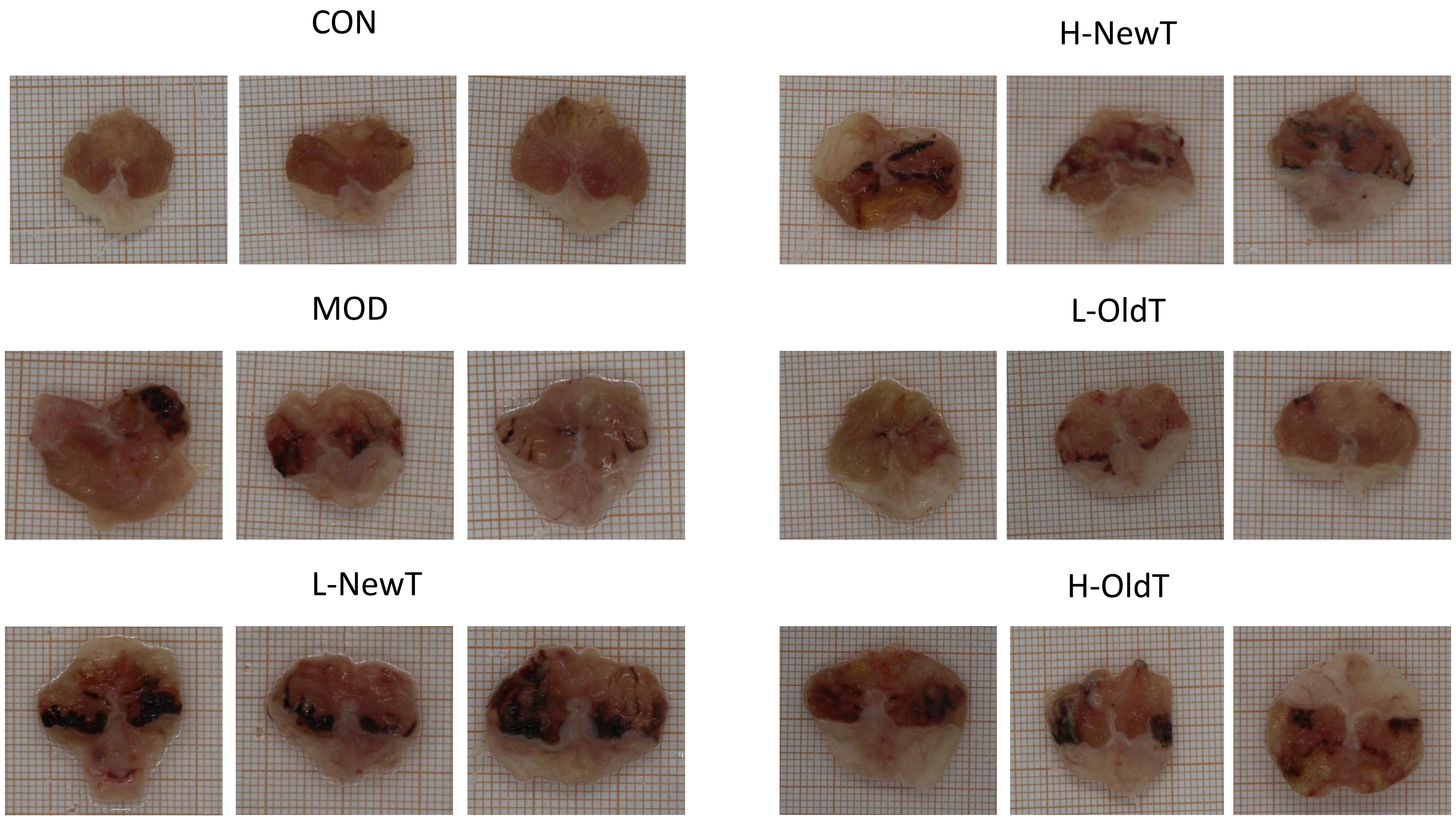

Supplement: Supplementary file 1 [file foods-14-02797-s001.zip › foods-3643990-supplementary.pdf]
